# Nurse-Led treatment strategy based on asthma score and oxygen requirement in children with acute reversible bronchoconstriction: A randomised controlled non-inferiority trial

**DOI:** 10.1371/journal.pone.0361579

**Published:** 2026-10-30

**Authors:** Sam Louman, Britt Foreman, Lidy van Lente, Richard Kop, Laura Postma, Mark Jan Ploegstra, Annemie L. M. Boehmer, Arvid W. A. Kamps

**Affiliations:** 1 Department of Paediatrics and Spaarne Gasthuis Academy, Spaarne Gasthuis, Haarlem, The Netherlands; 2 Department of Paediatrics, Frisius Medical Centre, Leeuwarden, The Netherlands; 3 Department of Clinical Epidemiology, Martini Academy, Martini Hospital, Groningen, The Netherlands; 4 Department of Paediatrics, Martini Hospital, Groningen, The Netherlands; Christian Medical College, INDIA

## Abstract

**Objective:**

The Childhood Asthma Score (CAS) does not fully account for oxygen therapy in children with acute reversible bronchoconstriction (ARB), thus the CAS O2 was developed. Our aim was to assess effectiveness and safety of the CAS O2 treatment protocol compared to the CAS protocol.

**Design:**

Multicentre, open-label, randomised, non-inferiority trial.

**Setting:**

Two Dutch teaching hospitals with general paediatric wards.

**Patients:**

Children 2–18 years admitted with ARB, requiring supplemental oxygen.

**Interventions:**

Nurse led treatment strategies using CAS O_2_ (tailored for patients receiving supplemental oxygen) or CAS to guide bronchodilator administration and reassessment intervals.

**Main outcome measure:**

Length of hospital stay (LOS); non-inferiority margin of 8 hours.

**Results:**

Data of 132 children who required supplemental oxygen were analysed (CAS O2 group n = 69; CAS n = 63). Median LOS was 40.0 hours (IQR 25.5–47.0) with CAS O_2_ and 40.8 hours (IQR 27.1–65.2) with CAS. The adjusted absolute difference in LOS (CAS O_2_ − CAS) was −3.7 hours (95% CI −10.2 to 4.1), meeting non inferiority. Bronchodilator administrations were fewer with CAS O2 (mean 7.9 versus 10.5; rate ratio 0.75, (95% CI 0.64–0.88; p < 0.001). Safety outcomes (physician assessments, intravenous magnesium sulphate or salbutamol, PICU transfer, 7 day readmission) did not differ between groups.

**Conclusions:**

Compared with CAS, CAS O_2_ supports nurse-led management of children hospitalised with ARB, including those requiring supplemental oxygen, with fewer bronchodilator administrations and no prolongation of LOS. These findings support broader implementation of CAS O2 -based pathways.

## Introduction

Acute reversible bronchoconstriction (ARB) due to acute viral wheeze or asthma is a leading cause of paediatric hospital admission [1]. Initial treatment comprises inhaled bronchodilators and systemic corticosteroids; intravenous magnesium sulphate and/or salbutamol are reserved for non-response. Bronchodilator administration in children with ARB is guided by clinical assessment. Multiple paediatric asthma scores standardise severity grading and correlate with clinical outcomes, but most include lung auscultation, which has variable reproducibility, particularly for wheeze, and requires specific training [2–9]. Clinical pathways, including nurse-led pathways, have been shown to reduce length of stay (LOS) in patients with asthma [4–8]. However, many rely on lung auscultation to assess bronchodilator requirements, requiring trained personnel. The Childhood Asthma Score (CAS) addressed this limitation by eliminating the need for auscultation, enabling nursing staff to independently assess bronchodilator requirements. Derived from the Qureshi score, CAS omits auscultatory findings while demonstrating good agreement with physicians’ bronchodilator decisions, as well as strong sensitivity, specificity, and inter-rater reliability [10,11]. Use of a CAS-based, nurse-led pathway has been shown to reduce LOS compared with historical auscultation-based care, with minimal need for physician consultation [12].

However, the CAS may overestimate the need for bronchodilator treatment in children requiring supplemental oxygen. In the original score, the use of supplemental oxygen automatically results in a minimum score of 4 points, which meets the threshold for bronchodilator administration, even in the absence of other signs of bronchoconstriction. This assumes that hypoxaemia primarily reflects bronchoconstriction, whereas it may also result from airway obstruction due to inflammation or mucus plugging. For example, mucus plugging can cause ventilation–perfusion mismatch or shunting, leading to hypoxaemia without significant bronchoconstriction. In these situations, bronchodilator therapy is unlikely to be effective, yet the original CAS continues to recommend treatment for as long as supplemental oxygen is required. To address this limitation, we developed CAS O_2_, which requires at least one additional sign of bronchoconstriction besides oxygen requirement before bronchodilator treatment is recommended.

We hypothesised that CAS O_2_ would be non-inferior to CAS with respect to LOS, while reducing bronchodilator use without evidence of harm.

The present study aimed to evaluate the feasibility and safety of a CAS O_2_-based, nurse-led treatment strategy compared with CAS in children hospitalised with ARB.

## Methods

### Trial design and setting

We conducted an open, multicentre, randomised, prospective, non-inferiority trial in two Dutch teaching hospitals. Both hospitals use a nurse-led CAS-based pathway as standard care. The Medical Ethics Review Committee of the University Medical Center Groningen (NL76447.042.21) and Institutional Review Boards (MEC 2021-037) approved the protocol. The trial was registered in the Dutch Clinical Trial Registry (https://www.onderzoekmetmensen.nl/nl/trial/50952). Participants were randomized using a computer-generated variable block randomization scheme, stratified by site, using Castor EDC.

### Participants

Eligible patients were children aged 2–18 years admitted to a general paediatric ward with acute reversible bronchoconstriction (ARB). ARB was defined as an acute episode of dyspnoea and wheeze, with suspected reversible airway obstruction, typically triggered by infection or environmental factors. Exclusion criteria were: very severe ARB requiring immediate intravenous salbutamol therapy; haemodynamically significant congenital or acquired heart disease or other clinically relevant cardiac abnormalities; chronic or congenital respiratory disease other than asthma; neurological or muscular comorbidity, or global developmental delay. Data were collected for enrolled patients who required supplemental oxygen during admission (Fig 1). Written informed consent was obtained from parents/caregivers and/or patients.

**Fig 1:**
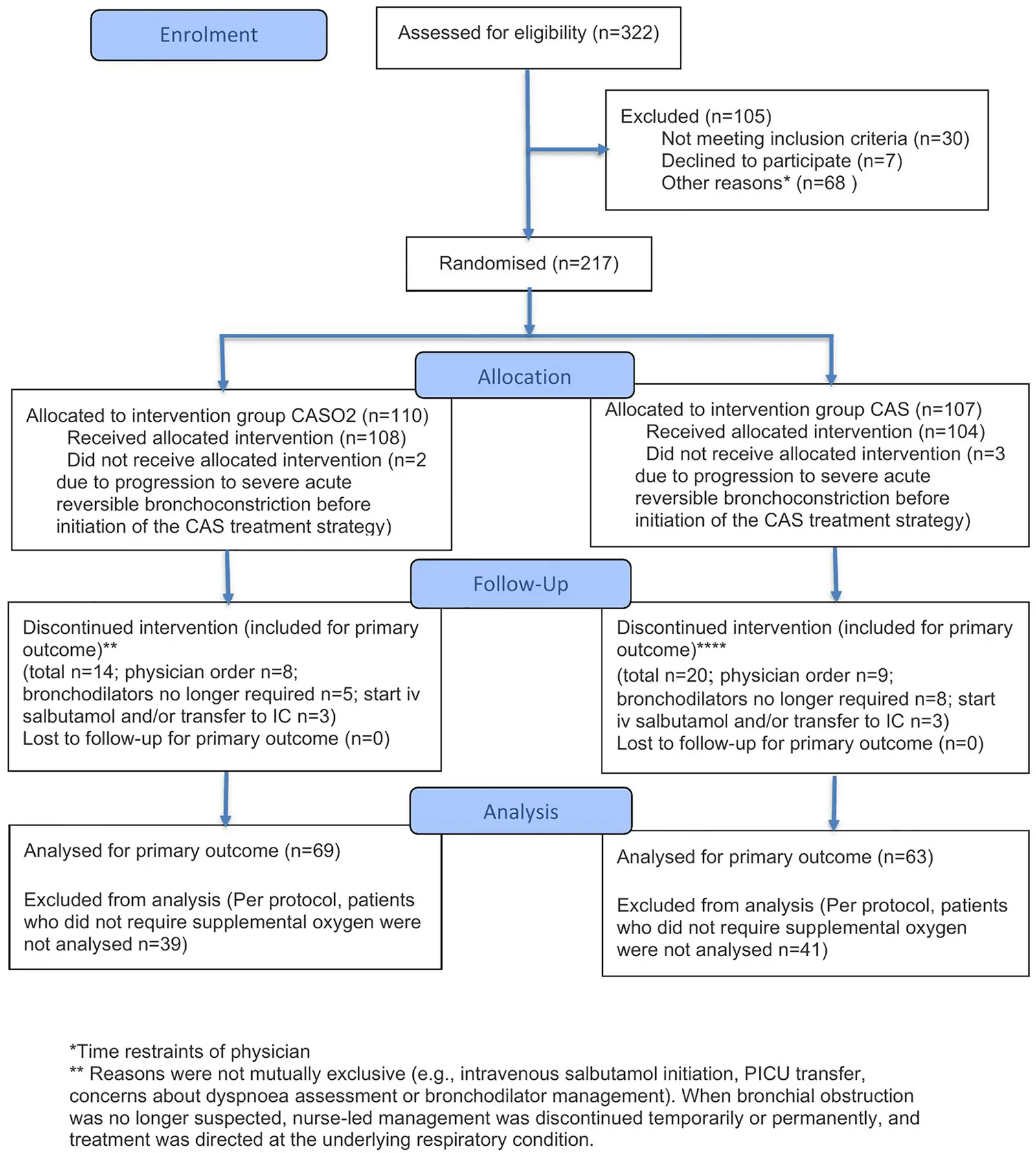
CONSORT Diagram. ARB: acute reversible bronchoconstriction. CAS: childhood asthma score.

### Trial procedures

Emergency department (ED) management during the first hour followed the Dutch Paediatric Society (NVK) acute asthma guideline [13]. Approximately one hour after presentation, physicians evaluated treatment response and decided on admission. After consent, patients were randomised to CAS or CAS O_2_. Those who declined were managed per CAS (usual care).

The nursing staff, pediatricians, and resident physicians attended educational sessions before implementation of the treatment strategy. Throughout the study period, the treatment strategy was regularly reinforced through clinical teaching sessions and newsletters. The local investigators were responsible for maintaining awareness of the study and treatment strategy among the clinical staff and were available for questions and clarification.

In both strategies, nurses assessed respiratory rate (score 1–3), peripheral oxygen saturation (SpO_2_; score 1–3), chest retractions (score 0–3), and dyspnoea (score 0–3). Individual scores were summed to yield a total score ranging from 2 to 12.

The total score guided decisions regarding physician consultation, bronchodilator administration, and the reassessment interval. CAS O_2_ applies different cut-offs for patients receiving supplemental oxygen (Fig 2). Nurses could request physician review at any time. If intravenous salbutamol therapy was initiated or transfer to the PICU was required, nurse-led management ceased and further care was directed by physicians. Autonomous registration of the CAS O_2_ score was defined as the nurse independently performing and recording the clinical assessment required to calculate the CAS O_2_ score, without involvement or intervention from the attending physician. “Bronchodilator no longer required” was defined as a low CAS O_2_ score on repeated assessment, indicating that no further bronchodilator treatment was required according to the treatment strategy. This needed to be confirmed by the attending physician based on the absence of clinical signs of bronchial obstruction on physical examination.

**Fig 2:**
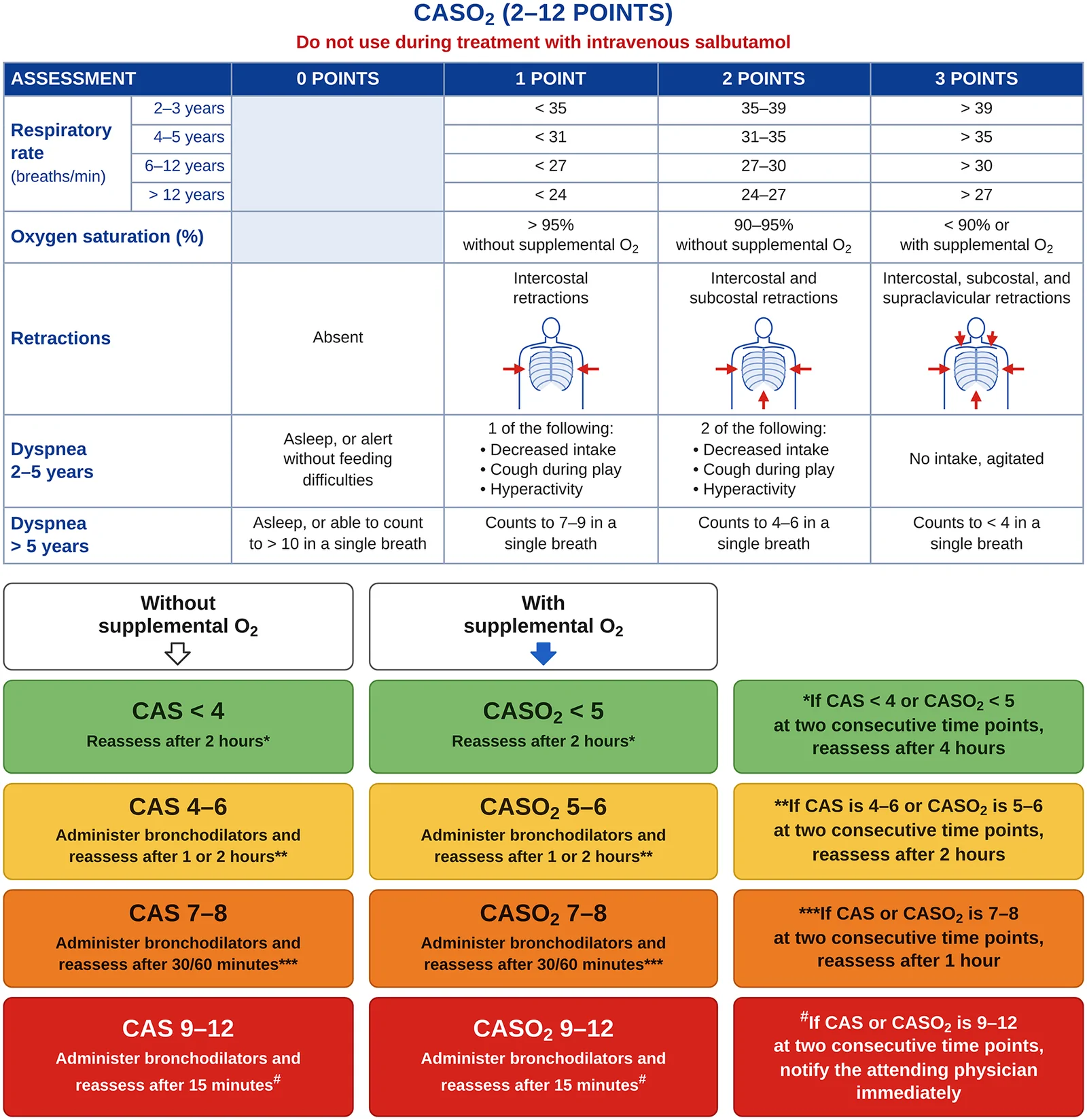
CAS O_2_ treatment strategy overview.

Other treatments, including systemic corticosteroids, intravenous therapy such as magnesium sulfate, and supplemental oxygen, were based on local clinical practice. Physicians assessed patients at least once daily during ward rounds, including auscultation. For this study, no predefined criteria were established for initiating or continuing inhaled corticosteroids after discharge. This decision was made by the treating physician based on the frequency of respiratory symptoms prior to the current admission and in accordance with local clinical practice.

### Outcomes

The primary outcome was LOS in hours. We prespecified an 8 hour non inferiority margin as clinically relevant because a difference <8 hours implies same day discharge. Secondary outcomes were: number of bronchodilator administrations per patient; deviations from the strategy (bronchodilators indicated but withheld; bronchodilators not indicated but given); duration of supplemental oxygen; and safety metrics (number of physician assessments; need for intravenous magnesium sulphate; need for intravenous salbutamol; PICU admission; readmission for ARB within 7 days).

### Sample size and statistical analysis

Based on pre-trial data from one of the participating hospitals, mean LOS is 42.7 hours (SD 18.3) [12]. Using these data, the prespecified 8-hour non-inferiority margin, a one-sided alpha of 0.05 and requiring 80% power, 130 patients (65 per group) requiring oxygen therapy were required.

We summarised nominal variables as counts (%), ordinal as medians (IQR), and continuous as means (SD) or medians (IQR) as appropriate. The primary result was the evaluation of non-inferiority against the pre-specified 8-hour margin. To perform this evaluation we used a linear mixed model fitted on log-transformed LOS, with random intercept for site and fixed effect for treatment, adjusted for sex, age, first Qureshi score at the ED, and pre-admission inhaled corticosteroid use. A log-linear model was used because LOS was right-skewed. The model gave the adjusted geometric mean ratio (GMR) with its 95% confidence interval, which were back-transformed to an adjusted absolute difference to evaluate non-inferiority. Both the GMR and back-transformed absolute difference were reported with their 95% confidence intervals.

The secondary outcome duration of supplemental oxygen was analysed using the same approach as the primary outcome. For the remaining secondary and safety outcomes: counts (bronchodilator administrations, physician assessments, number of CAS scores, treatment strategy deviations) were analysed with Poisson regression adjusted for number of assessments. Binary outcomes (need for other medication administrations, nurse-led treatment discontinuation, PICU admission and 7-day readmissions) were analysed with logistic regression.

We generated twenty imputed datasets for missing covariates and pooled estimates using Rubin’s rules. A complete‑case sensitivity analysis was performed for the primary outcome. All analyses were performed using R version 4.4.3 [14].

## Results

Between 23 January 2022 and 10 July 2025, 132 children were randomised: 69 to the CAS O_2_ group and 63 to the CAS group (Fig 1). Baseline characteristics were well balanced between groups (Table 1). Length of stay (LOS) data were complete for all participants. Covariate data were missing for 22 participants (17%; 13 (19%) in the CAS O_2_ group and 9 (14%) in the CAS group) and were handled using multiple imputation.

**Table 1:** Baseline characteristics.

	CAS O2 (n = 69)	CAS (n = 63)
Sex = Male	43 (62%)	40 (63%)
Age (yrs)	4.0 [3.0 - 5.9]	3.5 [2.7 - 5.6]
Qureshi asthma score at presentation, mean (SD)	n = 61 11.1 (2.1)	n = 50 11.0 (1.8)
Eczema	11 (16%)	13 (21%)
Allergic rhinitis	6 (9%)	8 (13%)
Medication use before admission		
Inhaled B2-agonist	50 (74%)	49 (78%)
Inhaled corticosteroids	20 (29%)	14 (22%)
Prednisolone	1 (1%)	0 (0%)
Antibiotics	5 (7%)	3 (5%)
Season of admission		
Spring	21 (30%)	15 (24%)
Summer	17 (25%)	18 (29%)
Autumn	20 (29%)	20 (32%)
Winter	10 (14%)	10 (16%)

Data are n (%), median (IQR) or mean (SD).

Median LOS was 40.0 hours (IQR 25.5–47.0) in the CAS O_2_ group and 40.8 hours (IQR 27.1–65.2) in the CAS group (Fig 3). The adjusted geometric mean ratio (GMR) for LOS was 0.91 (95% CI 0.75–1.09; p = 0.31). On the absolute scale, the adjusted difference in LOS (CAS O_2_ − CAS) was −3.7 hours (95% CI −10.2 to +4.1 hours). With an upper 95% confidence limit (+4.1 hours) well below the pre-specified non-inferiority margin of 8 hours, the primary outcome of non-inferiority was demonstrated. Results from the complete-case analysis were consistent, with an adjusted GMR of 0.90 (95% CI 0.73–1.11; p = 0.34) and an adjusted absolute difference of −4.0 hours (95% CI −11.0 to +4.7 hours).

**Fig 3:**
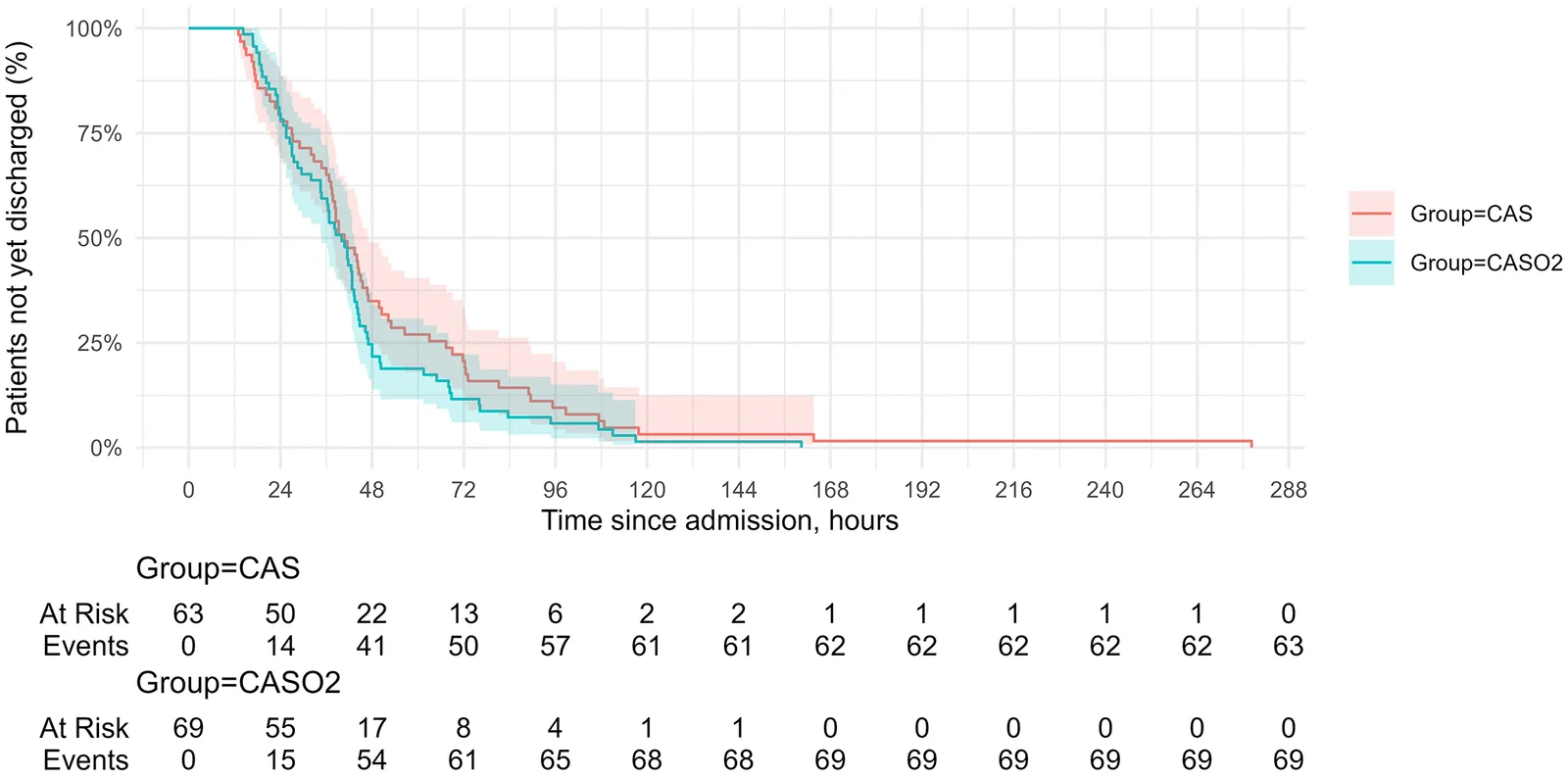
Patients not yet discharged over time per group. CAS: childhood asthma score.

Secondary outcomes are summarised in Table 2. Children managed with CAS O_2_ received fewer bronchodilator administrations than those managed with CAS (mean 7.9 vs 10.6 administrations per patient; rate ratio 0.75, 95% CI 0.70–0.90; p < 0.001). Deviations in which bronchodilators were indicated but withheld occurred less frequently in the CAS O_2_ group (rate ratio 0.42, 95% CI 0.26–0.67; p < 0.001), whereas deviations in which bronchodilators were administered despite no indication were similar between groups (rate ratio 0.81, 95% CI 0.54–1.33; p = 0.41). Nurses autonomously performed most assessments in both groups (88% [941/1068] in the CAS O_2_ group and 87% [856/985] in the CAS group). Duration of oxygen therapy was similar between groups.

**Table 2:** Secondary outcomes.

	CAS O2 (n = 69)	CAS (n = 63)	Adjusted effect estimate (95% CI)	p-value
Medication administered				
Systemic corticosteroids	68 (99%)	60 (95%)	3.8 [0.30–41.8]^1^	0.28
Duration, days	3.0 [3.0–3.0]	3.0 [3.0–3.0]	0.96 [0.91 - 1.02]^2^	0.19
Antibiotics	11 (16%)	9 (14%)	1.00 [0.34–2.97]^1^	1.00
MgSO_4_	12 (17%)	10 (16%)	1.19 [0.42 - 3.45]^1^	0.73
IV Salbutamol	3 (4%)	5 (8%)	0.53 [0.08–3.47]^1^	0.51
Number of CAS scores per patient	12.2 [10.0–16.0]	12.6 [7.9–17.2]	1.01 [0.92–1.13]^3^	0.87
Bronchodilator administrations	6.8 [4.0–10.1]	10.0 [5.2–13.4]	0.75 [0.65–0.88]^3^	< 0.001
Treatment strategy deviations				
Bronchodilators administered, when not advised	45/69 0.7 per patient	49/63 0.8 per patient	0.81 [0.50 – 1.33]^3^	0.41
Bronchodilators withheld, when advised to administer	29/69 0.4 per patient	65/63 1.0 per patient	0.42 [0.26–0.67]^3^	< 0.001
Physician assessments	127/69 1.8 per patient	129/63 2.1 per patient	0.89 [0.65–1.23]^3^	0.48
Nurse-led treatment discontinued	14 (20%)	20 (32%)	0.48 [0.20–1.14]^1^	0.09
Physician order	8 (12%)	9 (14%)	0.71 [0.24–2.16]^1^	0.55
Bronchodilators no longer required	5 (7%)	8 (13%)	0.46 [0.13–1.65]^1^	0.23
IV salbutamol administration	3 (4%)	5 (8%)	0.53 [0.08–3.47]^1^	0.51
PICU transfer	3 (4%)	5 (8%)	0.69 [0.10–4.84]^1^	0.71
Readmissions	0 (0%)	2 (3%)	NA*	NA*

95% CI: 95% Confidence interval. MgSO_4_: magnesiumsulfate. IV: intravenous. PICU: Pediatric Intensive Care Unit. ^1^Odds ratio. ^2^Geometric mean ratio. ^3^Rate ratio. *Model does not converge.

Safety outcomes were comparable between groups. The use of intravenous magnesium sulphate, intravenous salbutamol, PICU transfers, and 7-day readmissions did not differ. Nurse-led care was discontinued in 14 of 69 children (20%) in the CAS O_2_ group and 20 of 63 (32%) in the CAS group (odds ratio 0.48, 95% CI 0.20–1.14; p = 0.097). Reasons for discontinuation were comparable between groups. Inhaled corticosteroids were prescribed at discharge in 36 patients (52%) in the CAS O_2_ group and 30 patients (48%) in the CAS group (OR 1.02, 95% CI 0.46–2.25; p = 0.97).

## Discussion

In this multicentre randomised trial, a nurse-led treatment strategy using CAS O_2_ was non-inferior to a nurse-led treatment strategy using CAS with regard to LOS in children with ARB. In addition, CAS O_2_ reduced bronchodilator administrations without evidence of harm. These findings extend previous evidence demonstrating the benefits of nurse-led treatment pathways and of CAS and CAS O_2_ specifically [4–8,12].

Similar to other clinical pathways, a CAS-based nurse-led treatment strategy has previously been shown to reduce LOS [12]. CAS O_2_ is an optimised version with improved applicability in children requiring supplemental oxygen, and our findings suggest that comparable reductions in length of stay relative to physician-led care may also be achieved.

Besides demonstrating non-inferiority for LOS, children managed with CAS O_2_ received fewer bronchodilator administrations. This finding is consistent with the intended design of CAS O_2_, which avoids recommending bronchodilator treatment solely on the basis of supplemental oxygen requirement. Because hypoxaemia in acute asthma may also result from mechanisms other than bronchoconstriction, such as airway inflammation or mucus plugging, incorporating an additional sign of bronchoconstriction before treatment is recommended may reduce unnecessary bronchodilator use without adversely affecting clinical outcomes.

Considering the underlying pathophysiological mechanisms and the demonstrated non-inferiority regarding LOS, the reduction in bronchodilator administrations in the CAS O_2_ group suggests that a CAS O_2_-based treatment strategy may prevent unnecessary bronchodilator use. Importantly, nursing staff more frequently deviated from recommendations to administer bronchodilators in the CAS group, suggesting that the true difference in bronchodilator administrations between groups may be even greater.

Nurses performed nearly nine in ten assessments in both groups, confirming that a score without auscultation supports autonomous bedside decision-making. Although physician involvement was modestly higher than in our previous study with CAS, this likely reflects differences in case mix, including a higher proportion of children requiring continuous inhaled bronchodilator therapy or intravenous magnesium sulphate [12]. Although these findings suggest that nurse-led care may substantially reduce physician assessments, the extent of this reduction can only be determined in a direct comparison with physician-led care.

No between-group differences were observed in the use of intravenous magnesium sulphate, intravenous salbutamol, PICU transfer, or 7-day readmission rates. LOS itself may be considered a pragmatic safety indicator in ARB, as inadequate treatment is expected to lead to clinical deterioration, treatment escalation, and delayed discharge. The non-inferiority of LOS therefore aligns with the absence of differences in direct safety outcomes. Importantly, the reduction in bronchodilator administration did not prolong oxygen weaning or increase the frequency of physician reassessments, both of which could suggest undertreatment.

In a minority of patients (20–30%), nurse-led care was discontinued. In some cases, this was due to initiation of intravenous salbutamol or transfer to the PICU. Other reasons included physician concerns about accurately assessing dyspnoea severity, perceived overly long intervals between bronchodilator administrations, or uncertainty regarding continuation of bronchodilator therapy when the CAS O_2_ treatment strategy recommended withholding bronchodilators for more than 8 hours. In these situations, physicians evaluated whether ongoing bronchoconstriction was still present. If bronchial obstruction was no longer suspected, nurse-led management was discontinued temporarily or permanently, and treatment focused on the underlying respiratory condition. This reflects general paediatric practice, where respiratory diagnoses frequently overlap and bronchoconstriction may be transient, for example in children with lower respiratory tract infections. Importantly, nurse-led treatment strategies also appear feasible in this population while bronchoconstriction is present. However, reduced reliance on auscultation could theoretically delay discontinuation of bronchodilator therapy in some patients. Direct comparison with physician-led care is needed to further evaluate this potential limitation of nurse-led management.

Strengths of this trial include its randomised, pragmatic design and prespecified non-inferiority margin. In addition, the pathway was delivered by routine ward nurses under usual clinical conditions, supporting external applicability and implementation in daily practice.

Several limitations should also be considered. First, the open-label design may have influenced nurse and physician behaviour. Blinding was not feasible given the nature of the intervention; however, the study design reflects real-world implementation of both treatment strategies. Second, this trial directly compared CAS- and CAS O_2_-based care, limiting generalisability to centres where usual care remains physician-led and auscultation-based. Nevertheless, our findings demonstrate that CAS O_2_-based care does not increase LOS compared with CAS-based care, while previous studies have shown reduced LOS with CAS-based pathways relative to physician-led care. Future studies are desired to compare CAS O_2_ with physician-led, auscultation-based care across multiple centres and include both clinical and patient-centred outcomes to support broader implementation.

## Conclusions

Compared with CAS, CAS O_2_ supports nurse-led management of children hospitalised with ARB, including those requiring supplemental oxygen, with fewer bronchodilator administrations and no prolongation of LOS. Incorporating oxygen requirement into the treatment strategy may reduce overtreatment without delaying recovery and represents a pragmatic step towards more scalable and efficient paediatric acute respiratory care.
