# Supplementary material for: Nurse-Led treatment strategy based on asthma score and oxygen requirement in children with acute reversible bronchoconstriction: A randomised controlled non-inferiority trial
Source: PLoS One. 2026 Oct 30;21(10):e0361579. doi: 10.1371/journal.pone.0361579 (PMC13722160; doi:10.1371/journal.pone.0361579)
Supplement: Supplementary file 1 [file pone.0361579.s001.docx]

I would like to congratulate the authors on an excellent manuscript and commend them for the scientific rigor and robustness of the methodology. The study is well conducted and addresses an important clinical question. I have only a few comments and requests for clarification, as outlined below:

1. **Sample size calculation:** If available, could the authors consider using pre-trial LOS data (mean [SD]) from the participating institutions for the sample size calculation? Such data may provide a more accurate estimate of the expected variability in LOS and would make the sample size calculation more representative of the population under investigation.
2. **Figure labelling:** There appears to be a possible mislabelling of the figures. The authors are requested to review the figure numbering and corresponding references in the text and confirm that they are correct.
3. The manuscript states: *“Nurses autonomously performed most assessments in both groups (88% [941/1068] in the CAS O₂ group and 87% [856/985] in the CAS group).”* Please clarify what is meant by “autonomously performed” in this context and how these assessments were defined.
4. Approximately 50% of the children received inhaled corticosteroids (ICS) at discharge. Given that an asthma-related acute respiratory exacerbation would generally warrant initiation or continuation of controller therapy, it would be interesting to know the diagnostic profile of the enrolled children.
5. In Table 2, the meaning of the row **“Bronchodilator no longer required”** is unclear. Please clarify the operational definition of this outcome and how it was determined.
6. In future studies, it would be valuable to assess and report differences in readmission rates between the two groups. This would provide additional information regarding the longer-term clinical implications of the intervention.
