# Supplementary material for: Nurse-Led treatment strategy based on asthma score and oxygen requirement in children with acute reversible bronchoconstriction: A randomised controlled non-inferiority trial
Source: PLoS One. 2026 Oct 30;21(10):e0361579. doi: 10.1371/journal.pone.0361579 (PMC13722160; doi:10.1371/journal.pone.0361579)
Supplement: Supplementary file 2 [file pone.0361579.s003.docx]

**Response to reviewers**

We would like to thank the reviewers for their careful and constructive comments. We have carefully considered all comments and suggestions and have revised the manuscript accordingly. Below, we provide a point-by-point response to each comment. Changes made to the manuscript are indicated in the revised version.

**Reviewer #1:**

In methodology section, there is information about it being a multi cetric trial but it's not written about !. Number of sites, Location of sites?

*In the methods section it is stated that the trial was performed in two Dutch teaching hospitals.*

3. Whether any training was organised for all the nurses being participanrs/researchers in this trial to ensure uniformity in assessment and management?

*The nursing staff, pediatricians, and resident physicians attended educational sessions before implementation of the treatment strategy. Throughout the study period, the treatment strategy was regularly reinforced through clinical teaching sessions and newsletters. The local investigators were responsible for maintaining awareness of the study and treatment strategy among the clinical staff and were available for questions and clarification.*

4. The manuscript/ protocol has no clarity about uses of systemic cortico-steroids and IV therapies (other than albutorol) and supplemental oxygen. Whether sites had uniform policy or were variable?

*We have revised the following sentence in the method section as follows to clarify this point:*

*Other treatments, including systemic corticosteroids, intravenous therapy such as magnesium sulfate, and supplemental oxygen, were based on local clinical practice and applicable guidelines.*

**Reviewer #2:** I would like to congratulate the authors on an excellent manuscript and commend them for the scientific rigor and robustness of the methodology. The study is well conducted and addresses an important clinical question. I have only a few comments and requests for clarification, as outlined below:

1. Sample size calculation: If available, could the authors consider using pre-trial LOS data (mean [SD]) from the participating institutions for the sample size calculation? Such data may provide a more accurate estimate of the expected variability in LOS and would make the sample size calculation more representative of the population under investigation.

*We can confirm this was already the case. The mean and SD used in the sample size calculation (42.8 hours, SD 18.3) are taken from reference [12], a pre-trial study of LOS conducted at one of the two participating hospitals. This site went on to recruit the majority of patients in the present trial. We had not made this link explicit in the manuscript.*

*We have added a sentence to the Sample size paragraph clarifying that the mean and SD were derived from institutional pre-trial data: “Based on pre-trial data from one of the participating hospitals, mean LOS is 42.7 hours (SD 18.3) [12]. Using these data, the prespecified 8-hour non-inferiority margin, a one-sided alpha of 0.05 and requiring 80% power, 130 patients (65 per group) requiring oxygen therapy were required.”*

2. Figure labelling: There appears to be a possible mislabelling of the figures. The authors are requested to review the figure numbering and corresponding references in the text and confirm that they are correct.

*We carefully checked the labeling and numbering of all three figures, as well as their corresponding references in the text, and confirm that they are correct.*

3. The manuscript states: “Nurses autonomously performed most assessments in both groups (88% [941/1068] in the CAS O₂ group and 87% [856/985] in the CAS group).” Please clarify what is meant by “autonomously performed” in this context and how these assessments were defined.

*We agree with the reviewer that it is important to clarify what is meant by “autonomously performed” in this context. By “autonomously performed,” we mean that the assessment was performed independently by the nurse, without involvement or intervention from the attending physician.*

*We have clarified this definition in the methods section. “****Autonomous registration of the CAS O₂ score*** *was defined as the nurse independently performing and recording the clinical assessment required to calculate the CAS O₂ score, without involvement or intervention from the attending physician.”*

4. Approximately 50% of the children received inhaled corticosteroids (ICS) at discharge. Given that an asthma-related acute respiratory exacerbation would generally warrant initiation or continuation of controller therapy, it would be interesting to know the diagnostic profile of the enrolled children.

*We agree that the use of inhaled corticosteroids at discharge depends on the underlying diagnostic profile. In our practice, the decision to initiate or continue controller therapy was based on the frequency of respiratory symptoms prior to the current admission, as assessed by the attending physician.*

*We have clarified this in the method section by adding the following “For this study, no predefined criteria were established for initiating or continuing inhaled corticosteroids after discharge. This decision was made by the treating physician based on the frequency of respiratory symptoms prior to the current admission and in accordance with local clinical practice.”*

*As the prescription of inhaled corticosteroids at discharge was not a predefined secondary outcome, we have removed these data from Table 2. We now report the percentages descriptively at the end of the Results section instead, to provide information on discharge management.*

5. In Table 2, the meaning of the row “Bronchodilator no longer required” is unclear. Please clarify the operational definition of this outcome and how it was determined.

*This should indeed be clarified. By “bronchodilator no longer required,” we mean that repeated CAS O₂ assessments showed a low score for which, according to the treatment strategy, no further bronchodilator treatment was indicated, and that the consulting physician, based on the physical examination, concluded that there were no longer clinical signs of bronchial obstruction. We have added the definition of this outcome to the method section.*

*“Bronchodilator no longer required” was defined as a low CAS O₂ score on repeated assessment, indicating that no further bronchodilator treatment was required according to the treatment strategy, combined with confirmation by the consulting physician, based on physical examination, that there were no longer clinical signs of bronchial obstruction.*

6. In future studies, it would be valuable to assess and report differences in readmission rates between the two groups. This would provide additional information regarding the longer-term clinical implications of the intervention.

*Thank you for this valuable suggestion. We agree with the reviewer that monitoring readmission rates over a longer follow-up period would provide important information on the longer-term clinical implications of the intervention. We will consider this in future studies.*

**Reviewer #3:** Sample size: clarify that the alpha level used for the non-inferiority sample size calculation was one-sided. The assumptions used in the calculation, including the mean LOS, SD, and 8-hour non-inferiority margin, should be adequately justified. The cited paper does not specify the non-inferiority margin.

*We specified a one-sided alpha of 0.05. We realize using a one-sided alpha of 0.05 is less strict than the conventional 0.025. However, the upper bound of the 95% CI (+4.1 hours) remains well below the 8-hour non-inferiority margin, and the point estimate favors the intervention (LOS difference: -3.7 hours). Given this margin, we do not believe the less conservative alpha threatens the non-inferiority conclusion. Therefore, we have not listed this as a limitation but will add it if the reviewer prefers.".*

*LOS and SD stem from pre-trial LOS data from one of the participating institutions, for which reference 12 is cited. Indeed, the non-inferiority margin is not justified in reference 12, but is justified in the Outcomes paragraph in the Methods section: “We prespecified an 8 hour non inferiority margin as clinically relevant because a difference <8 hours implies same day discharge.” The sample size paragraph now reads as follows: “Based on pre-trial data from one of the participating hospitals, mean LOS is 42.7 hours (SD 18.3) [12]. Using these data, the prespecified 8-hour non-inferiority margin, a one-sided alpha of 0.05 and requiring 80% power, 130 patients (65 per group) requiring oxygen therapy were required.”*

The reported sample size calculation does not appear to correspond to the actual number of randomized participants (65 per arm vs 110 per arm were randomized). The sample size calculation should be an a priori calculation, rather than being derived post hoc based on the final number of participants available for analysis.

*The reported sample size calculation was performed a priori. Patients were recruited in the trial if admitted with acute reversible bronchoconstriction. But only those who required supplemental oxygen during admission were used for data collection. That is the sample the sample size calculation was aimed at. In the sample size calculation section (see previous answer) we have clarified that 65 patients requiring oxygen therapy per group are required.*

It appears that several statistical methods were used to analyze LOS. The authors needs to provide justification of why method was used. Do not present all available results without careful consideration. If non-inferiority is the primary objective, the non-inferiority analysis should be clearly treated as the primary analysis and should be the focus of the primary finding.

*We agree that it seems LOS was analyzed in multiple ways. We have now clarified how these ways are actually one total evaluation leading to the primary outcome: the evaluation of non-inferiority using an adjusted absolute difference. We have also clarified the results section to reflect this.*

*In Methods: “The primary result was the evaluation of non-inferiority against the pre-specified 8-hour margin. To perform this evaluation we used a linear mixed model fitted on log-transformed LOS, with random intercept for site and fixed effect for treatment, adjusted for sex, age, first Qureshi score at the ED, and pre-admission inhaled corticosteroid use. A log-linear model was used because LOS was right-skewed. The model gave the adjusted geometric mean ratio (GMR) with its 95% confidence interval, which were back-transformed to an adjusted absolute difference to evaluate non-inferiority. Both the GMR and back-transformed absolute difference were reported with their 95% confidence intervals.”*

*In Results: “The adjusted geometric mean ratio (GMR) for LOS was 0.91 (95% CI 0.75–1.09; p=0.31). On the absolute scale, the adjusted difference in LOS (CAS O₂ − CAS) was −3.7 hours (95% CI −10.2 to +4.1 hours). With an upper 95% confidence limit (+4.1 hours) well below the pre-specified non-inferiority margin of 8 hours, the primary outcome of non-inferiority was demonstrated.”*

The rationale for reporting the geometric mean ratio (GMR) should be provided.

*This is now included in the methods sections, see above.*

The Statistical Methods section should clearly specify which statistical method was used for each outcome or endpoint.

*We have included each outcome and endpoint in the methods and specified the statistical method used. This now corresponds with the footnotes in Table 2 as well.*

6. PLOS authors have the option to publish the peer review history of their article. If published, this will include your full peer review and any attached files.

If you choose “no”, your identity will remain anonymous but your review may still be made public.

**Do you want your identity to be public for this peer review?** For information about this choice, including consent withdrawal, please see our [Privacy Policy](https://urldefense.com/v3/__https:/track.editorialmanager.com/CL0/https:*2F*2Fwww.plos.org*2Fprivacy-policy/1/010f01a0323691f5-1e225aff-d20d-4890-b14b-b3264a038ee8-000000/928Vf_q0mZqgRVZvX4JCJlAdECLBLpC3LjtRVdH-Ipk=258__;JSUl!!IreeWePGmA!q0jwyY9z1jinK_3VJztuwmeTNpu3l7-lJuMY3bvIiJ6ci65MgpUbKODSxQ8DHQdPhA7eaP4R1WZpIxc$).

Reviewer #1: **Yes:** Prof (Dr.) Chandra Mohan Kumar

Reviewer #2: **Yes:** Nikita Agarwal

Reviewer #3: No
