# Supplementary material for: Nurse-Led treatment strategy based on asthma score and oxygen requirement in children with acute reversible bronchoconstriction: A randomised controlled non-inferiority trial
Source: PLoS One. 2026 Oct 30;21(10):e0361579. doi: 10.1371/journal.pone.0361579 (PMC13722160; doi:10.1371/journal.pone.0361579)
Supplement: Peer review history

# Peer review: Decision Letter 0

**DOI:** 10.1371/journal.pone.0361579.r001

**Authors:** George Kuryan

24 Aug 2026

Dear Dr. Kamps,

Thank you for submitting your manuscript to PLOS One. After careful consideration, we feel that it has merit but does not fully meet PLOS One’s publication criteria as it currently stands. Therefore, we invite you to submit a revised version of the manuscript that addresses the points raised during the review process.

Please submit your revised manuscript by Oct 23 2026 11:59PM. If you will need more time than this to complete your revisions, please reply to this message or contact the journal office at plosone@plos.org. When you're ready to submit your revision, log on to https://www.editorialmanager.com/pone/ and select the 'Submissions Needing Revision' folder to locate your manuscript file.

- A letter that responds to each point raised by the academic editor and reviewer(s). You should upload this letter as a separate file labeled 'Response to Reviewers'.
- A marked-up copy of your manuscript that highlights changes made to the original version. You should upload this as a separate file labeled 'Revised Manuscript with Track Changes'.
- An unmarked version of your revised paper without tracked changes. You should upload this as a separate file labeled 'Manuscript'.

If you would like to make changes to your financial disclosure, please include your updated statement in your cover letter. Guidelines for resubmitting your figure files are available below the reviewer comments at the end of this letter.

If applicable, we recommend that you deposit your laboratory protocols in protocols.io to enhance the reproducibility of your results. Protocols.io assigns your protocol its own identifier (DOI) so that it can be cited independently in the future. For instructions see: https://journals.plos.org/plosone/s/submission-guidelines#loc-laboratory-protocols. Additionally, PLOS One offers an option for publishing peer-reviewed Lab Protocol articles, which describe protocols hosted on protocols.io. Read more information on sharing protocols at https://plos.org/protocols?utm_medium=editorial-email&utm_source=authorletters&utm_campaign=protocols.

As the corresponding author, your ORCID iD is verified in the submission system and will appear in the published article. PLOS supports the use of ORCID, and we encourage all coauthors to register for an ORCID iD and use it as well. Please encourage your coauthors to verify their ORCID iD within the submission system before final acceptance, as unverified ORCID iDs will not appear in the published article. *Only*  the individual author can complete the verification step; PLOS staff *cannot*  verify ORCID iDs on behalf of authors.

We look forward to receiving your revised manuscript.

Kind regards,

George Kuryan

Academic Editor

PLOS One

**Journal Requirements:**

When submitting your revision, we need you to address these additional requirements.

1. Please ensure that your manuscript meets PLOS ONE's style requirements, including those for file naming. The PLOS ONE style templates can be found at

https://journals.plos.org/plosone/s/file?id=wjVg/PLOSOne_formatting_sample_main_body.pdf and

https://journals.plos.org/plosone/s/file?id=ba62/PLOSOne_formatting_sample_title_authors_affiliations.pdf

2. Please ensure that you have uploaded a completed CONSORT flowchart as part of your manuscript (typically figure 1). Blank copies of this document and information regarding CONSORT can be found via the following link: (https://www.consort-spirit.org/).

3. Thank you for stating the following in the Competing Interests section:

“I have read the journal's policy and the authors of this manuscript have the following competing interests: AK has received funding payed to institution for the present work from Martini Hospital Science Fund. SL, AB and AK have received funding payed to institution from ZonMw for a related trial utilizing CASO2. The authors have declared no other competing interests exist.”

Please confirm that this does not alter your adherence to all PLOS ONE policies on sharing data and materials, by including the following statement: "This does not alter our adherence to  PLOS ONE policies on sharing data and materials.” (as detailed online in our guide for authors http://journals.plos.org/plosone/s/competing-interests).  If there are restrictions on sharing of data and/or materials, please state these. Please note that we cannot proceed with consideration of your article until this information has been declared.

Please include your updated Competing Interests statement in your cover letter; we will change the online submission form on your behalf.

4. Please include captions for your Supporting Information files at the end of your manuscript, and update any in-text citations to match accordingly. Please see our Supporting Information guidelines for more information: http://journals.plos.org/plosone/s/supporting-information.

5. If the reviewer comments include a recommendation to cite specific previously published works, please review and evaluate these publications to determine whether they are relevant and should be cited. There is no requirement to cite these works unless the editor has indicated otherwise.

[Note: HTML markup is below. Please do not edit.]

Reviewers' comments:

Reviewer's Responses to Questions

**Comments to the Author**

1. Is the manuscript technically sound, and do the data support the conclusions?

Reviewer #1: Yes

Reviewer #2: Yes

Reviewer #3: Partly

**********

2. Has the statistical analysis been performed appropriately and rigorously?

Reviewer #1: Yes

Reviewer #2: Yes

Reviewer #3: No

**********

3. Have the authors made all data underlying the findings in their manuscript fully available?

The PLOS Data policy

Reviewer #1: Yes

Reviewer #2: Yes

Reviewer #3: Yes

**********

4. Is the manuscript presented in an intelligible fashion and written in standard English?

Reviewer #1: Yes

Reviewer #2: Yes

Reviewer #3: Yes

**********

Reviewer #1: In methodology section, there is information about it being a multi cetric trial but it's not written about !. Number of sites, Location of sites? 3. Whether any training was organised for all the nurses being participanrs/researchers in this trial to ensure uniformity in assessment and management?

4. The manuscript/ protocol has no clarity about uses of systemic cortico-steroids and IV therapies (other than albutorol) and supplemental oxygen. Whether sites had uniform policy or were variable?

Reviewer #2: I would like to congratulate the authors on an excellent manuscript and commend them for the scientific rigor and robustness of the methodology. The study is well conducted and addresses an important clinical question. I have only a few comments and requests for clarification, as outlined below:

1. Sample size calculation: If available, could the authors consider using pre-trial LOS data (mean [SD]) from the participating institutions for the sample size calculation? Such data may provide a more accurate estimate of the expected variability in LOS and would make the sample size calculation more representative of the population under investigation.

2. Figure labelling: There appears to be a possible mislabelling of the figures. The authors are requested to review the figure numbering and corresponding references in the text and confirm that they are correct.

3. The manuscript states: “Nurses autonomously performed most assessments in both groups (88% [941/1068] in the CAS O_2_ group and 87% [856/985] in the CAS group).” Please clarify what is meant by “autonomously performed” in this context and how these assessments were defined.

4. Approximately 50% of the children received inhaled corticosteroids (ICS) at discharge. Given that an asthma-related acute respiratory exacerbation would generally warrant initiation or continuation of controller therapy, it would be interesting to know the diagnostic profile of the enrolled children.

5. In Table 2, the meaning of the row “Bronchodilator no longer required” is unclear. Please clarify the operational definition of this outcome and how it was determined.

6. In future studies, it would be valuable to assess and report differences in readmission rates between the two groups. This would provide additional information regarding the longer-term clinical implications of the intervention.

Reviewer #3: Sample size: clarify that the alpha level used for the non-inferiority sample size calculation was one-sided. The assumptions used in the calculation, including the mean LOS, SD, and 8-hour non-inferiority margin, should be adequately justified. The cited paper does not specify the non-inferiority margin.

The reported sample size calculation does not appear to correspond to the actual number of randomized participants (65 per arm vs 110 per arm were randomized). The sample size calculation should be an a priori calculation, rather than being derived post hoc based on the final number of participants available for analysis.

It appears that several statistical methods were used to analyze LOS. The authors needs to provide justification of why method was used. Donot present all available results without careful consideration. If non-inferiority is the primary objective, the non-inferiority analysis should be clearly treated as the primary analysis and should be the focus of the primary finding.

The rationale for reporting the geometric mean ratio (GMR) should be provided.

The Statistical Methods section should clearly specify which statistical method was used for each outcome or endpoint.

**********

what does this mean?). If published, this will include your full peer review and any attached files.

If you choose “no”, your identity will remain anonymous but your review may still be made public.

**Do you want your identity to be public for this peer review?**  For information about this choice, including consent withdrawal, please see our Privacy Policy

Reviewer #1: **Yes:**  Prof (Dr.) Chandra Mohan Kumar

Reviewer #2: **Yes:**  Nikita Agarwal

Reviewer #3: No

**********

[NOTE: If reviewer comments were submitted as an attachment file, they will be attached to this email and accessible via the submission site. Please log into your account, locate the manuscript record, and check for the action link "View Attachments". If this link does not appear, there are no attachment files.]

To ensure your figures meet our technical requirements, please review our figure guidelines: https://journals.plos.org/plosone/s/figures

You may also use PLOS’s free figure tool, NAAS, to help you prepare publication quality figures: https://journals.plos.org/plosone/s/figures#loc-tools-for-figure-preparation.

NAAS will assess whether your figures meet our technical requirements by comparing each figure against our figure specifications.

Attachment Submitted filename: Reviewers Comments 20.docx

# Author comment: Author response to Decision Letter 1

**DOI:** 10.1371/journal.pone.0361579.r002

18 Sep 2026

Response to reviewers

We would like to thank the reviewers for their careful and constructive comments. We have carefully considered all comments and suggestions and have revised the manuscript accordingly. Below, we provide a point-by-point response to each comment. Changes made to the manuscript are indicated in the revised version.

Reviewer #1:

In methodology section, there is information about it being a multi cetric trial but it's not written about !. Number of sites, Location of sites?

In the methods section it is stated that the trial was performed in two Dutch teaching hospitals.

3. Whether any training was organised for all the nurses being participanrs/researchers in this trial to ensure uniformity in assessment and management?

The nursing staff, pediatricians, and resident physicians attended educational sessions before implementation of the treatment strategy. Throughout the study period, the treatment strategy was regularly reinforced through clinical teaching sessions and newsletters. The local investigators were responsible for maintaining awareness of the study and treatment strategy among the clinical staff and were available for questions and clarification.

4. The manuscript/ protocol has no clarity about uses of systemic cortico-steroids and IV therapies (other than albutorol) and supplemental oxygen. Whether sites had uniform policy or were variable?

We have revised the following sentence in the method section as follows to clarify this point:

Other treatments, including systemic corticosteroids, intravenous therapy such as magnesium sulfate, and supplemental oxygen, were based on local clinical practice and applicable guidelines.

Reviewer #2: I would like to congratulate the authors on an excellent manuscript and commend them for the scientific rigor and robustness of the methodology. The study is well conducted and addresses an important clinical question. I have only a few comments and requests for clarification, as outlined below:

1. Sample size calculation: If available, could the authors consider using pre-trial LOS data (mean [SD]) from the participating institutions for the sample size calculation? Such data may provide a more accurate estimate of the expected variability in LOS and would make the sample size calculation more representative of the population under investigation.

We can confirm this was already the case. The mean and SD used in the sample size calculation (42.8 hours, SD 18.3) are taken from reference [12], a pre-trial study of LOS conducted at one of the two participating hospitals. This site went on to recruit the majority of patients in the present trial. We had not made this link explicit in the manuscript.

We have added a sentence to the Sample size paragraph clarifying that the mean and SD were derived from institutional pre-trial data: “Based on pre-trial data from one of the participating hospitals, mean LOS is 42.7 hours (SD 18.3) [12]. Using these data, the prespecified 8-hour non-inferiority margin, a one-sided alpha of 0.05 and requiring 80% power, 130 patients (65 per group) requiring oxygen therapy were required.”

2. Figure labelling: There appears to be a possible mislabelling of the figures. The authors are requested to review the figure numbering and corresponding references in the text and confirm that they are correct.

We carefully checked the labeling and numbering of all three figures, as well as their corresponding references in the text, and confirm that they are correct.

3. The manuscript states: “Nurses autonomously performed most assessments in both groups (88% [941/1068] in the CAS O_2_ group and 87% [856/985] in the CAS group).” Please clarify what is meant by “autonomously performed” in this context and how these assessments were defined.

We agree with the reviewer that it is important to clarify what is meant by “autonomously performed” in this context. By “autonomously performed,” we mean that the assessment was performed independently by the nurse, without involvement or intervention from the attending physician.

We have clarified this definition in the methods section. “Autonomous registration of the CAS O_2_ score was defined as the nurse independently performing and recording the clinical assessment required to calculate the CAS O_2_ score, without involvement or intervention from the attending physician.”

4. Approximately 50% of the children received inhaled corticosteroids (ICS) at discharge. Given that an asthma-related acute respiratory exacerbation would generally warrant initiation or continuation of controller therapy, it would be interesting to know the diagnostic profile of the enrolled children.

We agree that the use of inhaled corticosteroids at discharge depends on the underlying diagnostic profile. In our practice, the decision to initiate or continue controller therapy was based on the frequency of respiratory symptoms prior to the current admission, as assessed by the attending physician.

We have clarified this in the method section by adding the following “For this study, no predefined criteria were established for initiating or continuing inhaled corticosteroids after discharge. This decision was made by the treating physician based on the frequency of respiratory symptoms prior to the current admission and in accordance with local clinical practice.”

As the prescription of inhaled corticosteroids at discharge was not a predefined secondary outcome, we have removed these data from Table 2. We now report the percentages descriptively at the end of the Results section instead, to provide information on discharge management.

5. In Table 2, the meaning of the row “Bronchodilator no longer required” is unclear. Please clarify the operational definition of this outcome and how it was determined.

This should indeed be clarified. By “bronchodilator no longer required,” we mean that repeated CAS O_2_ assessments showed a low score for which, according to the treatment strategy, no further bronchodilator treatment was indicated, and that the consulting physician, based on the physical examination, concluded that there were no longer clinical signs of bronchial obstruction. We have added the definition of this outcome to the method section.

“Bronchodilator no longer required” was defined as a low CAS O_2_ score on repeated assessment, indicating that no further bronchodilator treatment was required according to the treatment strategy, combined with confirmation by the consulting physician, based on physical examination, that there were no longer clinical signs of bronchial obstruction.

6. In future studies, it would be valuable to assess and report differences in readmission rates between the two groups. This would provide additional information regarding the longer-term clinical implications of the intervention.

Thank you for this valuable suggestion. We agree with the reviewer that monitoring readmission rates over a longer follow-up period would provide important information on the longer-term clinical implications of the intervention. We will consider this in future studies.

Reviewer #3: Sample size: clarify that the alpha level used for the non-inferiority sample size calculation was one-sided. The assumptions used in the calculation, including the mean LOS, SD, and 8-hour non-inferiority margin, should be adequately justified. The cited paper does not specify the non-inferiority margin.

We specified a one-sided alpha of 0.05. We realize using a one-sided alpha of 0.05 is less strict than the conventional 0.025. However, the upper bound of the 95% CI (+4.1 hours) remains well below the 8-hour non-inferiority margin, and the point estimate favors the intervention (LOS difference: -3.7 hours). Given this margin, we do not believe the less conservative alpha threatens the non-inferiority conclusion. Therefore, we have not listed this as a limitation but will add it if the reviewer prefers.".

LOS and SD stem from pre-trial LOS data from one of the participating institutions, for which reference 12 is cited. Indeed, the non-inferiority margin is not justified in reference 12, but is justified in the Outcomes paragraph in the Methods section: “We prespecified an 8 hour non inferiority margin as clinically relevant because a difference <8 hours implies same day discharge.” The sample size paragraph now reads as follows: “Based on pre-trial data from one of the participating hospitals, mean LOS is 42.7 hours (SD 18.3) [12]. Using these data, the prespecified 8-hour non-inferiority margin, a one-sided alpha of 0.05 and requiring 80% power, 130 patients (65 per group) requiring oxygen therapy were required.”

The reported sample size calculation does not appear to correspond to the actual number of randomized participants (65 per arm vs 110 per arm were randomized). The sample size calculation should be an a priori calculation, rather than being derived post hoc based on the final number of participants available for analysis.

The reported sample size calculation was performed a priori. Patients were recruited in the trial if admitted with acute reversible bronchoconstriction. But only those who required supplemental oxygen during admission were used for data collection. That is the sample the sample size calculation was aimed at. In the sample size calculation section (see previous answer) we have clarified that 65 patients requiring oxygen therapy per group are required.

It appears that several statistical methods were used to analyze LOS. The authors needs to provide justification of why method was used. Do not present all available results without careful consideration. If non-inferiority is the primary objective, the non-inferiority analysis should be clearly treated as the primary analysis and should be the focus of the primary finding.

We agree that it seems LOS was analyzed in multiple ways. We have now clarified how these ways are actually one total evaluation leading to the primary outcome: the evaluation of non-inferiority using an adjusted absolute difference. We have also clarified the results section to reflect this.

In Methods: “The primary result was the evaluation of non-inferiority against the pre-specified 8-hour margin. To perform this evaluation we used a linear mixed model fitted on log-transformed LOS, with random intercept for site and fixed effect for treatment, adjusted for sex, age, first Qureshi score at the ED, and pre-admission inhaled corticosteroid use. A log-linear model was used because LOS was right-skewed. The model gave the adjusted geometric mean ratio (GMR) with its 95% confidence interval, which were back-transformed to an adjusted absolute difference to evaluate non-inferiority. Both the GMR and back-transformed absolute difference were reported with their 95% confidence intervals.”

In Results: “The adjusted geometric mean ratio (GMR) for LOS was 0.91 (95% CI 0.75–1.09; p=0.31). On the absolute scale, the adjusted difference in LOS (CAS O_2_ − CAS) was −3.7 hours (95% CI −10.2 to +4.1 hours). With an upper 95% confidence limit (+4.1 hours) well below the pre-specified non-inferiority margin of 8 hours, the primary outcome of non-inferiority was demonstrated.”

The rationale for reporting the geometric mean ratio (GMR) should be provided.

This is now included in the methods sections, see above.

The Statistical Methods section should clearly specify which statistical method was used for each outcome or endpoint.

We have included each outcome and endpoint in the methods and specified the statistical method used. This now corresponds with the footnotes in Table 2 as well.

________________________________________

6. PLOS authors have the option to publish the peer review history of their article. If published, this will include your full peer review and any attached files.

If you choose “no”, your identity will remain anonymous but your review may still be made public.

Do you want your identity to be public for this peer review? For information about this choice, including consent withdrawal, please see our Privacy Policy.

Reviewer #1: Yes: Prof (Dr.) Chandra Mohan Kumar

Reviewer #2: Yes: Nikita Agarwal

Reviewer #3: No

Attachment Submitted filename: Response to Reviewers.docx

# Peer review: Decision Letter 1

**DOI:** 10.1371/journal.pone.0361579.r003

**Authors:** George Kuryan

12 Oct 2026

Nurse-Led treatment strategy based on asthma score and oxygen requirement in children with acute reversible bronchoconstriction: a randomised controlled non-inferiority trial

PONE-D-26-35581R1

Dear Dr. Kamps

We’re pleased to inform you that your manuscript has been judged scientifically suitable for publication and will be formally accepted for publication once it meets all outstanding technical requirements.

Within one week, you’ll receive an e-mail detailing the required amendments. When these have been addressed, you’ll receive a formal acceptance letter and your manuscript will be scheduled for publication.

An invoice will be generated when your article is formally accepted. Please note, if your institution has a publishing partnership with PLOS and your article meets the relevant criteria, all or part of your publication costs will be covered. Please make sure your user information is up-to-date by logging into Editorial Manager at Editorial Manager® and clicking the ‘Update My Information' link at the top of the page. For questions related to billing, please contact billing support.

If your institution or institutions have a press office, please notify them about your upcoming paper to help maximize its impact. If they’ll be preparing press materials, please inform our press team as soon as possible -- no later than 48 hours after receiving the formal acceptance. Your manuscript will remain under strict press embargo until 2 pm Eastern Time on the date of publication. For more information, please contact onepress@plos.org.

Kind regards,

George Kuryan

Academic Editor

PLOS One

Additional Editor Comments (optional):

Reviewers' comments:

Reviewer's Responses to Questions

**Comments to the Author**

Reviewer #2: All comments have been addressed

Reviewer #3: All comments have been addressed

**********

2. Is the manuscript technically sound, and do the data support the conclusions?

Reviewer #2: Yes

Reviewer #3: (No Response)

**********

3. Has the statistical analysis been performed appropriately and rigorously?

Reviewer #2: Yes

Reviewer #3: (No Response)

**********

4. Have the authors made all data underlying the findings in their manuscript fully available?

The PLOS Data policy

Reviewer #2: Yes

Reviewer #3: (No Response)

**********

5. Is the manuscript presented in an intelligible fashion and written in standard English?

Reviewer #2: Yes

Reviewer #3: (No Response)

**********

Reviewer #2: Please add the response to the last comment about the comparison of re-admission data in the future studies section.

Reviewer #3: (No Response)

**********

what does this mean?). If published, this will include your full peer review and any attached files.

If you choose “no”, your identity will remain anonymous but your review may still be made public.

**Do you want your identity to be public for this peer review?**  For information about this choice, including consent withdrawal, please see our Privacy Policy

Reviewer #2: **Yes:**  Nikita Agarwal

Reviewer #3: No

**********

# Editor report: Acceptance letter

**DOI:** 10.1371/journal.pone.0361579.r004

**Authors:** George Kuryan

PONE-D-26-35581R1

PLOS One

Dear Dr. Kamps,

I'm pleased to inform you that your manuscript has been deemed suitable for publication in PLOS One. Congratulations! Your manuscript is now being handed over to our production team.

At this stage, our production department will prepare your paper for publication. This includes ensuring the following:

* All references, tables, and figures are properly cited

* All relevant supporting information is included in the manuscript submission,

* There are no issues that prevent the paper from being properly typeset

You will receive further instructions from the production team, including instructions on how to review your proof when it is ready. Please keep in mind that we are working through a large volume of accepted articles, so please give us a few days to review your paper and let you know the next and final steps.

Lastly, if your institution or institutions have a press office, please let them know about your upcoming paper now to help maximize its impact. If they'll be preparing press materials, please inform our press team within the next 48 hours. Your manuscript will remain under strict press embargo until 2 pm Eastern Time on the date of publication. For more information, please contact onepress@plos.org.

You will receive an invoice from PLOS for your publication fee after your manuscript has reached the completed accept phase. If you receive an email requesting payment before acceptance or for any other service, this may be a phishing scheme. Learn how to identify phishing emails and protect your accounts at https://explore.plos.org/phishing.

If we can help with anything else, please email us at customercare@plos.org.

Thank you for submitting your work to PLOS One and supporting open access.

Kind regards,

PLOS One Editorial Office Staff

on behalf of

Professor George Kuryan

Academic Editor

PLOS One
